# Safety profile and clinical activity of multiple subcutaneous doses of MEDI-528, a humanized anti-interleukin-9 monoclonal antibody, in two randomized phase 2a studies in subjects with asthma

**DOI:** 10.1186/1471-2466-11-14

**Published:** 2011-02-28

**Authors:** Joseph M Parker, Chad K Oh, Craig LaForce, S David Miller, David S Pearlman, Chenxiong Le, Gabriel J Robbie, Wendy I White, Barbara White, Nestor A Molfino

**Affiliations:** 1 MedImmune, LLC, Gaithersburg, MD, USA; 2 North Carolina Clinical Research, Raleigh, NC, USA; 3 Northeast Medical Research Associates, North Dartmouth, MA, USA; 4 Colorado Allergy & Asthma Centers PC, Denver, CO, USA; 5 Current address: UCB Biosciences, Inc., Raleigh, NC, USA

## Abstract

**Background:**

Interleukin-9 (IL-9)-targeted therapies may offer a novel approach for treating asthmatics. Two randomized placebo-controlled studies were conducted to assess the safety profile and potential efficacy of multiple subcutaneous doses of MEDI-528, a humanized anti-IL-9 monoclonal antibody, in asthmatics.

**Methods:**

Study 1: adults (18-65 years) with mild asthma received MEDI-528 (0.3, 1, 3 mg/kg) or placebo subcutaneously twice weekly for 4 weeks. Study 2: adults (18-50 years) with stable, mild to moderate asthma and exercise-induced bronchoconstriction received 50 mg MEDI-528 or placebo subcutaneously twice weekly for 4 weeks. Adverse events (AEs), pharmacokinetics (PK), immunogenicity, asthma control (including asthma exacerbations), and exercise challenge test were evaluated in study 1, study 2, or both.

**Results:**

In study 1 (N = 36), MEDI-528 showed linear serum PK; no anti-MEDI-528 antibodies were detected. Asthma control: 1/27 MEDI-528-treated subjects had 1 asthma exacerbation, and 2/9 placebo-treated subjects had a total of 4 asthma exacerbations (one considered a serious AE). In study 2, MEDI-528 (n = 7) elicited a trend in the reduction in mean maximum decrease in FEV_1 _post-exercise compared to placebo (n = 2) (-6.49% MEDI-528 vs -12.60% placebo; -1.40% vs -20.10%; -5.04% vs -15.20% at study days 28, 56, and 150, respectively). Study 2 was halted prematurely due to a serious AE in an asymptomatic MEDI-528-treated subject who had an abnormal brain magnetic resonance imaging that was found to be an artifact on further evaluation.

**Conclusions:**

In these studies, MEDI-528 showed an acceptable safety profile and findings suggestive of clinical activity that support continued study in subjects with mild to moderate asthma.

**Trial registration:**

ClinicalTrials (NCT): NCT00507130 and ClinicalTrials (NCT): NCT00590720

## Background

Asthma continues to be a significant health problem [1], with nearly 8% of the US population reported to have asthma in 2006 [2]. In one study, approximately 30% of >3,400 asthmatics failed to achieve control despite regular use of combination therapy with high-dose inhaled corticosteroids (ICS) and long-acting β_2_-agonists [3].

Interleukin (IL)-9, a 144 amino acid-long protein secreted by CD4^+ ^T-helper 2 (Th2) cells, mast cells, eosinophils, and neutrophils [4-7], may be associated with airway hyperresponsiveness (AHR) and inflammation [8-11]. Evidence supporting IL-9 as a potential target treatment for asthma emerged from a series of genetic experiments linking AHR to a region on chromosome 13 in mice, which contains the IL-9 gene and is syntenic with the 5q31-q33 chromosome in humans [8].

Overexpression of IL-9 in murine models of asthma has been shown to cause airway inflammation with pulmonary infiltration of eosinophils and lymphocytes, airway obstruction, and mast cell hyperplasia [9,10,12]. In contrast, anti−IL-9 antibody therapy has led to reduced levels of AHR in murine models of allergen-induced asthma [13,14].

Blocking IL-9 expression inhibits airway inflammation in a mast cell-dependent murine model of asthma. Mast cell-deficient animals demonstrated reduced lung inflammation and AHR compared with wild-type control mice [15]. An IL-9-neutralizing monoclonal antibody effectively reduced lung recovery of mast cell precursors and inflammatory cells after allergen challenge [16]. These findings suggest that IL-9 promotes asthma pathology in a mast cell-dependent manner through the proliferation of mast cell precursors or the recruitment of immature mast cells to lung tissue, or both.

Mast cell degranulation and release of spasmogenic mediators have been reported to cause bronchoconstriction in subjects with exercise-induced asthma [17,18]. Exercise challenge is an indirect airway challenge that results in airway narrowing due to the release of mediators from mast cell degranulation, as opposed to direct airway challenges such as methacholine that act directly on the airway smooth muscle to produce bronchoconstriction [19].

Additionally, in asthmatics, bronchial biopsy specimens revealed increased IL-9 immunoreactive cells and IL-9 mRNA, protein, and receptor levels compared with those of healthy controls [20-23]. These data suggest that IL-9-targeted therapies may offer a novel approach for treating patients with asthma and may reduce exercise-induced bronchoconstriction (EIB).

MEDI-528 is a humanized anti-IL-9 monoclonal antibody. Results from 2 open-label, phase 1 studies demonstrated that MEDI-528, administered as a single intravenous or subcutaneous (SC) dose, had an acceptable safety profile in healthy volunteers, with no serious adverse events (AEs) and a linear pharmacokinetic (PK) profile [24].

We report the results of 2 studies evaluating the safety, tolerability, PK, and immunogenicity profiles of multiple SC doses of MEDI-528, and the potential reduction of EIB in subjects with mild to moderate asthma. Study 2 was halted prematurely due to a serious AE (SAE) in an asymptomatic MEDI-528-treated subject who had an abnormal brain magnetic resonance imaging (MRI) that was found to be an artifact on further evaluation.

## Methods

### Subjects

Adults aged 18-65 years with mild persistent asthma (forced expiratory volume in 1 second [FEV_1_] or peak expiratory flow [PEF] ≥80% of predicted) receiving therapy with short-acting β_2_-agonists (SABA), inhaled corticosteroids (ICS) <264 μg/day fluticasone or equivalent, or both (study 1) and adults aged 18-50 years with stable mild to moderate persistent asthma receiving therapy with SABA, ICS <800 μg/day budesonide or equivalent, and EIB (decrease in FEV_1 _of ≥15% from baseline during screening) (study 2) were eligible [25].

Exclusion criteria included lung disease other than asthma, use of systemic immunosuppressive drugs, and smoking history ≥10 pack-years. Long-acting β_2_-agonists, cromolyn sodium, nedocromil sodium, leukotriene receptor antagonists, theophylline, and omalizumab were not allowed (studies 1 and 2).

### Study design

Study 1 was a randomized, double-blind, placebo-controlled, dose-escalation, multicenter study evaluating the safety, tolerability, PK, and immunogenicity profiles of multiple SC doses of MEDI-528. For each cohort (0.3 mg/kg, 1 mg/kg, or 3 mg/kg), subjects were randomized 3:1 via an interactive voice response system (IVRS) to receive MEDI-528 or placebo as SC injections twice weekly for 4 weeks through study day 24; thereafter, subjects were monitored for 126 days. Dosing at each next higher dose group commenced after all evaluable subjects from the previous lower dose group completed evaluations on study day 56 with acceptable safety profiles. Subjects who received ≥7 doses of the study drug were considered evaluable. Those not evaluable were replaced, unless they withdrew from the study due to safety reasons. The primary outcome for this study was the safety and tolerability of multiple SC doses of MEDI-528. Secondary outcomes included PK and immunogenecity of MEDI-528 in this subject population. Exploratory outcomes included effects of MEDI-528 on pulmonary function, asthma exacerbations, symptoms, rescue SABA use, and quality of life.

Study 2 was a randomized, double-blind, placebo-controlled, multicenter study evaluating the safety and tolerability profiles of multiple SC doses of MEDI-528 in three cohorts of 50, 100, and 200 mg versus placebo. Subjects were randomized 2:1 via IVRS to receive MEDI-528 or placebo as an SC injection twice weekly for 4 weeks; thereafter, subjects were monitored for 126 days. Subjects who received ≥4 doses of study drug (unless they discontinued for safety reasons) and had ≥2 exercise challenge tests (baseline and post-therapy) were considered evaluable. The primary outcome of this study was the safety and tolerability of multiple SC doses of MEDI-528 in adult subjects with stable asthma and EIB. Secondary objectives included the effect of MEDI-528 on EIB and immunogenicity. Exploratory outcomes included the effects of MEDI-528 on spirometry, airway hyperresponsiveness as measured by methacholine challenge testing, asthma exacerbations, asthma symptoms, rescue SABA use, quality of life, and nasal allergy symptoms in this population.

In both studies, all subjects and protocol-associated personnel were blinded to the individual subject treatment assignment until the last subject in each cohort completed the study and the databases were locked.

Both studies were conducted in accordance with the Declaration of Helsinki and were approved by an institutional review board/independent ethics committee at each participating site. Written informed consent was obtained from each subject before study entry.

### Safety profile

In both studies, AEs and SAEs were monitored after the first dose through day 150. AEs were graded by severity (mild, moderate, severe) and relationship to study drug (none, remote, possible, probable, definite) as determined by each investigator. Other safety measures included routine laboratory tests, vital signs, electrocardiograms (ECGs), and physical examinations. Physical examination included assessments for splenomegaly (palpable spleen), lymphadenopathy, and neurologic abnormalities.

In both studies, a noncontrast MRI of the brain was performed at screening and day 28. MRI was added to the current studies and other MEDI-528 studies [24] based on preclinical toxicology findings of lymphohistiocytic perivascular infiltrates in the brains of cynomolgus monkeys seen in both treated and control animals. The study and peer-review pathologists considered this a spontaneous background finding unrelated to treatment. Subsequent MEDI-528 toxicology studies in monkeys and of MM9C1 in mice found no evidence of macroscopic or microscopic pathologic changes in the brain; MRI is therefore not required for subsequent clinical studies of MEDI-528 (data on file, MedImmune, LLC).

Subjects in both studies who received any dose of study drug were included in the safety analyses.

### Pharmacokinetics and immunogenicity

In study 1, blood samples for measuring serum concentrations of MEDI-528 were collected before dosing and at specified times throughout the study. As previously described [24], a validated enzyme-linked immunosorbent assay (ELISA) was used for these measurements. Unknown values with calculated concentrations below the assay's lower limit of quantitation (< 1.25 μg/mL) were reported as less than the limit of quantitation.

A double-antigen sandwich ELISA was performed to evaluate anti-MEDI-528 antibodies [24].

### Excercise challenge test

In study 2, exercise challenge was performed at baseline prior to dosing and on study days 28, 56, and 150 after dosing [26]. A response to therapy was defined as a maximum decrease in post-exercise FEV_1 _of <10%, based on American Thoracic Society guidelines [27]. Spirometry was performed 15 minutes before initiation of the treadmill test and 5, 10, 15, 20, and 30 minutes after the treadmill test was completed. At each time point, maximum FEV_1 _was determined. The decrease in FEV_1 _at each time point after the treadmill test was calculated as a percentage of the best baseline FEV_1_.

### Asthma control and quality of life

In both studies, an asthma exacerbation was defined as a worsening of asthma requiring oral corticosteroids, a doubling of the ICS dose from baseline, hospitalization, emergency department visit, or an unscheduled asthma-related visit to a health care provider. Asthma symptom scores were recorded twice daily for the duration of the study. Symptoms were assessed on a scale of 0 (no symptoms) to 4 (marked discomfort). Rescue SABA use (puffs/day) was recorded daily by subjects for the duration of the study. Subjects completed the Asthma Quality of Life Questionnaire (AQLQ) [28] at baseline and after dosing.

### Additional evaluations

Skin prick testing of common food and aeroallergens was performed during screening for both studies and on study day 28 for study 1 [29]. In both studies, spirometry was performed according to existing guidelines [30].

### Statistical analyses

Formal sample size calculations were not applicable for assessment of the primary objective (safety/tolerability profile). No statistical hypothesis testing was performed for this end point. Data analyses were conducted using the SAS System (SAS Institute Inc., Cary, NC).

AEs and SAEs were described using the MedDRA Adverse Event Thesaurus by system organ class, severity, and relationship to study drug through 18 weeks after the last dose (day 150). Laboratory values with a higher toxicity grade than that observed at baseline were recorded as AEs. The day 0 value before study drug administration was used as a baseline for laboratory parameters. Study discontinuation blood samples were summarized at the closest nominal time point that did not already have a value.

The original design for study 2 included 3 cohorts (50 mg, 100 mg, and 200 mg MEDI-528), each with 18 subjects randomized 2:1 to receive MEDI-528 or placebo. Sample size calculations were based on two-sample *t *test of the reduction in the maximum decline of FEV_1 _after exercise challenge testing. The power to detect a statistically significant difference in the maximum decline in FEV_1 _was greater than 80% based on an assumption of a 20% fall in the placebo group and 65% reduction in the maximum decline of FEV_1 _in the combined group (36 subjects) on active treatment versus placebo (18 subjects).

Serum concentrations and PK parameters were analyzed using WinNonlin (Pharsight, St. Louis, MO) and descriptive statistics summarized for each MEDI-528 treatment group. The number of subjects exhibiting anti-MEDI-528 antibodies was summarized, and all valid assay results from subjects who received any study drug were included in immunogenicity summaries.

No formal statistical hypothesis tests were conducted for exploratory variables (pulmonary function, asthma exacerbations, symptom scores, rescue SABA use, quality of life). These variables were examined for their medical/clinical implications.

Two-sample *t *tests were used to explore the change from baseline in FEV_1 _and asthma symptom score between MEDI-528 and placebo groups. The Fisher exact test was used to explore the difference in asthma exacerbation proportions between the placebo and MEDI-528 groups. The total number of exacerbations per subject during the study was noted.

## Results

### Demographics and baseline characteristics

**Figure 1:**
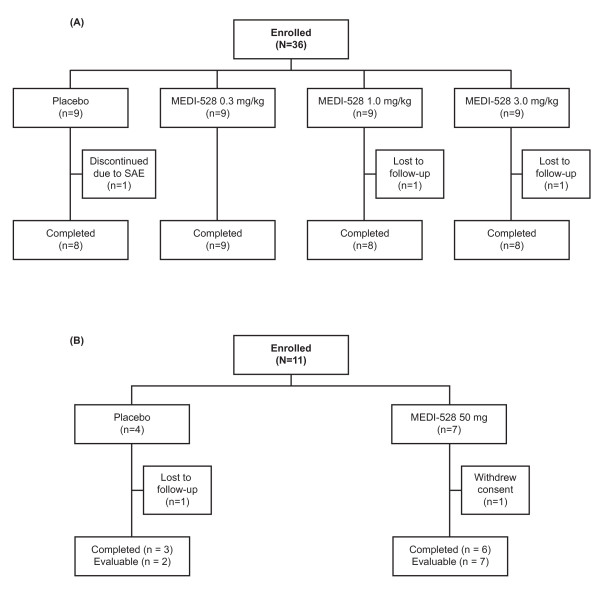
**Flow diagram for subjects in studies 1 (A) and 2 (B)**.

In study 1, 36 subjects were randomized between June 2007 and February 2008 at 8 sites, and 33 completed the study through day 150. Two MEDI-528-treated subjects were lost to follow-up and 1 placebo-treated subject withdrew due to an asthma exacerbation requiring hospitalization. All 36 subjects were evaluable (ie, received ≥7 doses of study drug) and included in the safety analyses (Figure 1A). There were more ex-smokers in the placebo group, otherwise the groups' baseline characteristics were comparable (Table 1).

**Table 1:** Demographics and Baseline Characteristics (ITT Population)

	Study 1				Study 2	
			
		MEDI-528				
		
Parameter	Placebo (n = 9)	0.3 mg/kg (n = 9)	1 mg/kg (n = 9)	3 mg/kg (n = 9)	Placebo (n = 4)	MEDI-528 50 mg (n = 7)
Mean age, years (range)	38.6 (22-63)	38.2 (18-61)	33.2 (19-57)	28.9 (19-55)	31.8 (19-47)	32.1 (19-39)
Female, n (%)	8 (88.9)	8 (88.9)	5 (55.6)	5 (55.6)	2 (50)	6 (85.7)
Race/Ethnicity, n (%)						
White/non-Hispanic	8 (88.9)	6 (66.7)	9 (100)	5 (55.6)	3 (75.0)	6 (85.7)
White/Hispanic	0 (0.0)	0 (0.0)	0 (0.0)	0 (0.0)	0 (0.0)	1 (14.3)
Black	0 (0.0)	2 (22.2)	0 (0.0)	3 (33.3)	1 (25.0)	0 (0.0)
Asian	0 (0.0)	0 (0.0)	0 (0.0)	1 (11.1)	0 (0.0)	0 (0.0)
Multiracial	1 (11.1)	1 (11.1)	0 (0.0)	0 (0.0)	0 (0.0)	0 (0.0)
Mean weight, kg (range)	68.5 (52.0-93.9)	78.9 (58.2-99.7)	72.3 (43.0-99.1)	72.7 (57.6-97.7)	77.3 (57.7-93.2)	73.4 (52.7-97.3)
Mean (SD) FEV_1 _(L)*	2.8 (0.58)	2.8 (0.93)	3.2 (0.73)	3.4 (0.43)	3.3 (1.25)	2.8 (0.56)
Ever smoked, n (%)	7 (77.8)	1 (11.1)	1 (11.1)	0 (0.0)	1 (25.0)	2 (28.6)
Exacerbations in past year, no. (%)						
None	4 (44.4)	6 (66.7)	6 (66.7)	4 (44.4)	2 (50.0)	3 (42.9)
1-2	5 (55.6)	3 (33.3)	2 (22.2)	5 (55.6)	2 (50.0)	4 (57.1)
3-5	0 (0.0)	0 (0.0)	1 (11.1)	0 (0.0)	0 (0.0)	0 (0.0)

*Measurements from evaluable subjects (those who received ≥7 or ≥4 doses of the study drug in study 1 and study 2, respectively); ITT = intent to treat (all randomized subjects); SD = standard deviation; FEV_1 _= forced expiratory volume in 1 second.

In study 2, 11 subjects were randomized and dosed with 50 mg of MEDI-528 or placebo between February and May 2008 at 4 sites, and 9 completed the study through day 150 (Figure 1B). Nine subjects were evaluable (ie, received ≥4 doses of study drug). Two placebo-treated subjects were not evaluable (1 received only 2 doses of study drug and 1 was lost to follow-up). All 11 subjects were included in the safety analyses. Baseline characteristics were similar between study groups (Table 1).

### Safety profile

The most frequently reported AEs in study 1 are listed in Table 2. Severe AEs were reported in 4 placebo-treated subjects (vomiting, n = 1; elevated lipase, n = 2; asthma, n = 1) and 2 subjects receiving MEDI-528 0.3 mg/kg (severe diarrhea, n = 1; elevated alanine aminotranferase [ALT], n = 1). The severe asthma AE in the 1 placebo-treated subject was an SAE (asthma exacerbation requiring hospitalization); this subject was discontinued from the study. The elevated ALT seen in the MEDI-528-treated subject was noted on the first day of dosing. This subject received no further drug and the elevated levels resolved by study day 42. No deaths were noted during this study.

**Table 2:** Most Frequently Reported Adverse Events in Study 1 (Safety Population*)

	No. (%) of Subjects				
	
Event		MEDI-528			
		
	Placebo (n = 9)	0.3 mg/kg (n = 9)	1 mg/kg (n = 9)	3 mg/kg (n = 9)	Total (n = 27)
Blood glucose increase	3 (33.3)	5 (55.6)	2 (22.2)	1 (11.1)	8 (29.6)
Nasopharyngitis	4 (44.4)	3 (33.3)	1 (11.1)	2 (22.2)	6 (22.2)
Blood bicarbonate decrease	1 (11.1)	1 (11.1)	2 (22.2)	3 (33.3)	6 (22.2)
Injection site pain	0 (0.0)	3 (33.3)	0 (0.0)	3 (33.3)	6 (22.2)
Pharyngolaryngeal pain	1 (11.1)	2 (22.2)	3 (33.3)	0 (0.0)	5 (18.5)
Lymphocyte count decrease	0 (0.0)	0 (0.0)	4 (44.4)	1 (11.1)	5 (18.5)
Injection site bruising	1 (11.1)	0 (0.0)	1 (11.1)	3 (33.3)	4 (14.8)
Presence of protein in urine	1 (11.1)	2 (22.2)	1 (11.1)	1 (11.1)	4 (14.8)
Vomiting	3 (33.3)	0 (0.0)	1 (11.1)	1 (11.1)	2 (7.4)
Urinary tract infection	2 (22. 2)	1 (11.1)	0 (0.0)	1 (11.1)	2 (7.4)
Presence of blood in urine	1 (11.1)	1 (11.1)	1 (11.1)	0 (0.0)	2 (7.4)
Blood chloride increase	2 (22.2)	1 (11.1)	0 (0.0)	0 (0.0)	1 (3.7)
Blood potassium decrease	2 (22.2)	0 (0.0)	1 (11.1)	0 (0.0)	1 (3.7)
Influenza	2 (22.2)	0 (0.0)	1 (11.1)	0 (0.0)	1 (3.7)
Lipase increase	2 (22.2)	1 (11.1)	0 (0.0)	0 (0.0)	1 (3.7)
Sinusitis	2 (22.2)	0 (0.0)	1 (11.1)	0 (0.0)	1 (3.7)

Values are shown in descending order of frequency in the total MEDI-528 group.

*Consisted of all subjects who received the study drug.

Abnormal but clinically nonsignificant ECG results were detected during the study in 5 placebo-treated subjects, 5 subjects in the MEDI-528 0.3-mg/kg group, and 2 subjects each in the MEDI-528 1-mg/kg and 3-mg/kg groups. One subject in the MEDI-528 3-mg/kg group exhibited an asymptomatic elevation of troponin levels at a single time point on study day 84. No significant changes in the central nervous system were observed from the brain MRI or focused neurological examinations.

The most frequently reported AEs in study 2 are listed in Table 3. A total of 5 severe AEs occurred in 4 subjects; 3 placebo-treated subjects had 1 event each (eye infection, cough, drug hypersensitivity) and 1 MEDI-528-treated subject had 2 events (sunburn, back pain). No significant ECG changes occurred and no elevations in troponin levels were observed. One SAE occurred in a MEDI-528-treated subject (abnormal brain MRI results). The subject had a 6- × 4-mm left-sided pontine hyperintensity noted on the day 28 MRI that was not present at baseline. The investigator considered the event possibly related to study drug, resulting in a clinical hold of the study. A repeat MRI with gadolinium contrast showed no abnormal findings or pontine hyperintensity. Review by an independent neuroradiologist determined the initial MRI finding to be an artifact. The clinical hold was lifted, but the study was discontinued due to the length of the delay.

**Table 3:** Most Frequently Reported Adverse Events in Study 2 (Safety Population*)

	No. (%) of Subjects	
	
Event	Placebo (n = 4)	MEDI-528 50 mg (n = 7)
Blood glucose increased	0 (0.0)	2 (28.6)
Back pain	1 (25.0)	1 (14.3)
Injection site irritation	1 (25.0)	1 (14.3)
Blood bicarbonate increased	0 (0.0)	1 (14.3)
Cyst	0 (0.0)	1 (14.3)
Lipase increased	0 (0.0)	1 (14.3)
Lower respiratory tract infection	0 (0.0)	1 (14.3)
Nuclear MRI abnormal	0 (0.0)	1 (14.3)

Values are shown in descending order of frequency in the MEDI-528 group. MRI = magnetic resonance imaging.

*Consisted of all subjects who received the study drug.

### Pharmacokinetics and immunogenicity

In study 1, limited PK parameters were estimable because the dosing interval for MEDI-528 was not constant, alternating between 3 days and 4 days, and PK sampling was sparse.

After the last MEDI-528 dose, maximum serum concentrations were generally achieved between 3 and 4 days across dose levels (Table 4). Mean maximum concentration after the last dose of 0.3 mg/kg to 3 mg/kg, respectively, increased in an approximately dose-proportional manner from 13.7 μg/mL to 105.5 μg/mL; similar dose proportionality was noted for trough concentrations, which increased from 11.7 μg/mL to 90.4 μg/mL. Mean half-life was similar across dose levels (range, 35-38 days).

**Table 4:** MEDI-528 Multiple-Dose Pharmacokinetic Parameters in Study 1

Cohort	C_max_ (μg/mL)	T_max_ (day)	T_1/2_ (day)	Accumulation index*
0.3 mg/kg (n = 9)	13.7 ± 2.7^†^	3.5 ± 2.1^†^	37.1 ± 7.5^† ^	5.2 ± 2.0^‡^
1 mg/kg (n = 9)	52.1 ± 33.0	3.7 ± 2.3	35.0 ± 11.5	9.9 ± 4.5
3 mg/kg (n = 9)	105.5 ± 31.0	3.9 ± 2.6	37.7 ± 7.5	6.7 ± 1.8

Values are mean ± SD. C_max _= maximum concentration; T_max _= time to maximum concentration; T_1/2 _= half-life.

*Accumulation index was based on trough concentration after first and last dose.

^†^n = 8.

^‡^n = 7.

**Figure 2:**
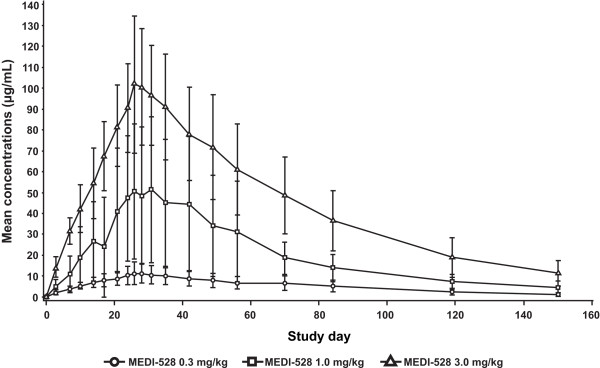
**MEDI-528 mean serum concentrations in study 1**. Mean maximum concentration after the last dose of 0.3 mg/kg to 3 mg/kg, respectively, mean half-life, trough concentrations after the first and last doses were measured. Mean concentrations of MEDI-528 increased in a dose-proportional manner and peaked after the last dose of the study drug.

Comparison of trough concentrations after the first and last doses yielded accumulation index values between 5 and 10 across dose levels. The fluctuation of MEDI-528 concentrations within a dosing interval was small, consistent with the frequency of dosing and half-life (Figure 2).

No anti-MEDI-528 antibodies, defined as antibody titer of >10, were detected in any group during study 1 or study 2.

### Pulmonary function

Pulmonary function was generally unchanged throughout both studies. FEV_1 _values were comparable among groups at baseline and at the end of the studies (Table 5). In study 1, FEV_1 _percent predicted values were comparable at baseline and end of study (Table 5).

**Table 5:** Exploratory Analyses in Studies 1 and 2 (Evaluable Population)

	Study 1				Study 2	
			
		MEDI-528				
		
Parameter	Placebo	0.3 mg/kg	1 mg/kg	3 mg/kg	Placebo	MEDI-528 50 mg
Pulmonary function						
FEV_1_, L						
Baseline	2.8 ± 0.58 (n = 9)	2.8 ± 0.93 (n = 9)	3.2 ± 0.73 (n = 9)	3.4 ± 0.43 (n = 9)	3.3 ± 1.25 (n = 2)	2.8 ± 0.56 (n = 7)
End of study	2.9 ± 0.48 (n = 8)	2.6 ± 1.02 (n = 9)	3.3 ± 0.67 (n = 8)	3.6 ± 0.56 (n = 8)	3.0 ± 1.30 (n = 2)	2.9 ± 0.42 (n = 5)
FEV_1_, % predicted						
Baseline	89.3 ± 10.49 (n = 9)	85.8 ± 7.95 (n = 9)	86.0 ± 12.07 (n = 9)	93.3 ± 12.40 (n = 9)		
End of study	91.4 ± 8.87 (n = 8)	79.2 ± 11.04 (n = 9)	90.3 ± 9.64 (n = 8)	99.6 ± 13.14 (n = 8)		
Asthma symptom score, overall						
Baseline	1.11 ± 0.83 (n = 6)	1.87 ± 1.70 (n = 7)	1.37 ± 1.59 (n = 6)	0.00 ± N/A (n = 1)	1.92 ± 1.53 (n = 2)	0.93 ± 0.82 (n = 6)
Treatment	0.74 ± 0.55 (n = 7)	0.91 ± 0.63 (n = 6)	1.10 ± 1.49 (n = 6)	0.13 ± 0.18 (n = 2)	0.91 ± 0.07 (n = 2)	0.41 ± 0.66 (n = 6)
Rescue short-acting β_2_-agonist use (no. of puffs/day), overall during study	0.63 ± 0.60 (n = 9)	0.65 ± 0.51 (n = 8)	0.66 ± 0.80 (n = 8)	0.02 ± 0.04 (n = 5)	0.22 ± 0.02 (n = 2)	0.28 ± 0.32 (n = 7)
AQLQ score, overall						
Baseline	5.56 ± 1.11 (n = 9)	5.20 ± 0.79 (n = 9)	5.73 ± 0.70 (n = 9)	5.97 ± 0.78 (n = 9)	5.73 ± 0.11 (n = 2)	5.75 ± 0.65 (n = 7)
Day 28	5.64 ± 0.82 (n = 9)	5.68 ± 0.66 (n = 9)	5.95 ± 0.87 (n = 9)	6.07 ± 0.60 (n = 9)	6.22 ± 0.09 (n = 2)	5.94 ± 0.57 (n = 7)

Values are mean ± SD.

AQLQ = Asthma Quality of Life Questionnaire; FEV_1 _= forced expiratory volume in 1 second.

### Asthma control and quality of life

Asthma control and quality of life results are shown in Table 5. In both studies, a trend toward improvement in the overall mean asthma symptom scores was noted during the treatment period in all subjects compared with baseline values. Overall mean rescue SABA use during both studies was comparable among groups, and overall mean AQLQ scores were comparable between the MEDI-528 groups and placebo at baseline and during the study.

In study 1, fewer subjects having ≥1 asthma exacerbation were observed in the combined MEDI-528 group (n = 1 of 27) compared with the placebo group (n = 2 of 9; *P *= 0.148). The one MEDI-528-treated subject was receiving the lowest dose. The 2 placebo-treated subjects had a total of 4 asthma exacerbation episodes (1 subject had 1 episode; 1 subject had 3 episodes).

In study 2, no asthma exacerbations were reported.

### Exercise challenge test

**Figure 3:**
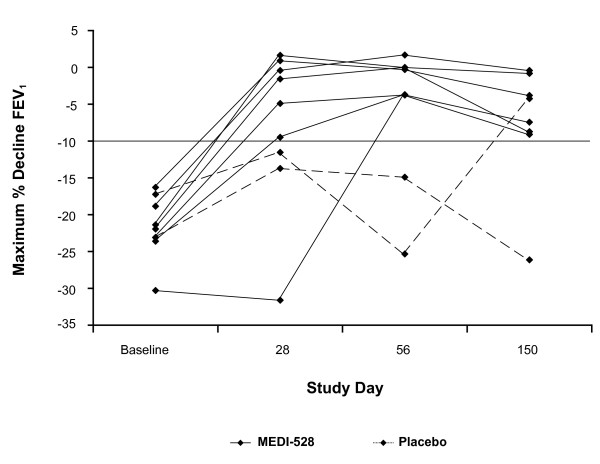
**Mean maximum percentage decline in FEV_1 _after exercise for each individual subject in study 2**. Multiple doses of MEDI-528 resulted in a reduction in the mean maximum percentage decrease in FEV_1 _after exercise as compared to placebo.

In study 2, multiple doses of MEDI-528 resulted in a reduction in the mean maximum percentage decrease in FEV_1 _after exercise as compared to placebo (Table 6). The mean absolute maximum decline in FEV_1 _at study day 56 was -0.04 L for the MEDI-528 group compared with -0.60 L for the placebo group (*P *< 0.01). Differences at all other data points did not achieve statistical significance (data not shown). Time to return to 90% of baseline FEV_1 _following exercise challenge was shorter during the screening period prior to dosing with study drug and at all subsequent study days for the MEDI-528 group as compared with placebo. Furthermore, the time to return to 90% of baseline FEV_1 _improved in the MEDI-528 group at study days 28 and 56, while there was no improvement in the placebo group. A post hoc analysis showed that 6 of 7 MEDI-528-treated subjects were responders at study day 28, 7 of 7 at day 56, and 6 of 6 at day 150. There were no placebo-treated responders at days 28 and 56, and there was 1 responder at day 150 (Figure 3).

**Table 6:** Mean (SD) maximum percentage change in FEV_1 _after exercise in study 2.

	**Baseline**	***P *Value**	**Day 28**	***P *Value**	**Day 56**	***P *Value**	**Day 150**	***P *Value**
	
MEDI-528 (n = 7)*	-22.20 (4.38)	0.62	-6.49 (11.73)	0.22	-1.40 (2.27)	0.17	-5.04 (3.91)	0.52
Placebo (n = 2)	-20.20 (4.16)		-12.60 (1.53)		-20.10 (7.41)		-15.20 (15.48)	

*For day 150, n = 6. *P *values are based on two-sample *t *test between placebo and MEDI-528 groups.

## Discussion

In both studies, subjects tolerated multiple SC doses of MEDI-528, with AE, severe AE and SAE rates similar to placebo-treated subjects. No subjects developed anti-MEDI-528 antibodies. MEDI-528 exhibited linear PK, with peak and trough concentrations increasing in an approximately dose-proportional manner over the dose range studied. The half-life values (35-38 days) were consistent with those observed in the high-dose groups of the single-dose SC study of healthy volunteers [24], namely, the 3-mg/kg (half-life, 44 days) and 9-mg/kg (half-life, 33 days) doses. In addition, these results are in accord with findings from the 2 studies conducted in healthy volunteers [24]. In those studies, MEDI-528 had an acceptable safety profile, with no SAEs or deaths reported.

Throughout study 1, pulmonary function was essentially unchanged and SABA use was comparable among groups. Overall, asthma symptom scores showed a trend toward improvement in all subjects. A trend toward fewer subjects having ≥1 asthma exacerbation was observed in MEDI-528-treated subjects in study 1 compared with placebo-treated subjects. This finding is of unclear significance as blocking IL-9 may result in a reduction in AHR [13,14] and AHR has not been clearly related to asthma exacerbations.

Although the actions of IL-9 are not fully understood, IL-9 is believed to play an important role in the trafficking and function of mast cells [15,16]. Local IL-9 production from inflammatory cells would theoretically result in the recruitment and differentiation of mast cell progenitors from the bone marrow to the lung. Blocking IL-9 would therefore not be expected to have an immediate clinical effect, but this would rather be dependent on the loss of resident mast cells in the tissue.

The results of study 2 are intriguing in that they suggest that blocking IL-9 with MEDI-528 may have an effect on EIB, which is dependent on mast cell degranulation [19]. The maximum effect of MEDI-528 was seen at study day 56, which would be consistent with a later onset of action. Future studies should consider that a period of chronic dosing may be required before seeing maximum clinical effect. Conclusions from study 2 are unfortunately limited in that it was prematurely halted.

Because of the small sample size in both studies, firm conclusions regarding the clinical activity of MEDI-528 cannot be drawn from these results. The potential benefits of MEDI-528 observed in both studies should be interpreted with caution as these studies were also limited by the mild disease severity of the study population.

## Conclusions

In conclusion, prior and current results suggest that MEDI-528 has acceptable safety and tolerability profiles and provide evidence of clinical activity in mild to moderate asthmatics. Further studies are warranted to assess the clinical efficacy of MEDI-528 for treating patients with inadequately controlled asthma.

## Competing interests

CLF, SDM, and DP received research funding from MedImmune, LLC, for the conduct of these studies. JMP, CKO, CL, GJR, WIW and NAM are employees of MedImmune, LLC. BW was an employee of MedImmune, LLC, at the time of the study and manuscript submission. These studies were sponsored by MedImmune, LLC.

## Authors' contributions

CLF, SDM, and DP were involved in the collection of data and interpretation of the results; JMP, CKO, CL, GJR, WIW, BW, and NAM were involved in the design of the study, analysis of the data, and interpretation of the results; all authors critically reviewed and revised the manuscript and approved the final version.

## Pre-publication history

The pre-publication history for this paper can be accessed here:

http://www.biomedcentral.com/1471-2466/11/14/prepub
